# Spontaneous Bacterial Peritonitis in Cardiogenic Ascites

**DOI:** 10.7759/cureus.10995

**Published:** 2020-10-16

**Authors:** Muhammad Usman Zafar, Jasraj Marjara, Zahid Ijaz Tarar, Ghulam Ghous, Preysi Patel

**Affiliations:** 1 Hospital Medicine, Lehigh Valley Health Network, Allentown, USA; 2 Internal Medicine, University of Missouri, Columbia, USA; 3 Hematology/Oncology, University of Missouri, Columbia, USA; 4 Internal Medicine, Lehigh Valley Health Network, Allentown, USA

**Keywords:** spontaneous bacterial peritonitis, cardiac ascites, saag, constrictive pericarditis, pericardiectomy, paracentesis

## Abstract

The most common cause of ascites is liver cirrhosis. Additional causes such as heart failure, cancer, and pancreatitis among others can also precipitate this abnormality. Spontaneous bacterial peritonitis (SBP) is an infection of ascitic fluid that happens without any evidence of an intra-abdominal surgically-treatable cause. Ascites of cardiac origin can also be complicated by SBP. Here we present a case of a 62-year-old male with extensive cardiac history who presented to our service with ongoing dyspnea and orthopnea. He also had significant abdominal distention and pitting edema. The patient was found to have constrictive pericarditis and was admitted for pericardiectomy. Ascitic fluid was consistent with a transudative process. Lab and imaging did not show evidence of liver or kidney disease. Ascitic fluid was indicative of ascites of cardiac origin. Postoperatively patient developed intermittent fevers initially thought to be due to pericarditis but later found to be due to SBP complicating his recurrent ascites. Such a temporal association of SBP that complicates ascites after pericardiectomy has not been discussed frequently in literature.

## Introduction

Ascites is defined as a collection of more than 25 mL of fluid in the peritoneal cavity. The most common cause is liver cirrhosis. Additional causes include heart failure, cancer, pancreatitis, tuberculosis, and hepatic vein obstruction [1]. Of these causes, cardiogenic ascites from heart failure only contributes to 5% of total ascites cases. Ascites can be commonly complicated by spontaneous bacterial peritonitis (SBP). SBP is defined as an infection of the ascitic fluid that occurs without evidence of an intra-abdominal surgically-treatable source [2]. This infection frequently occurs in the presence of hepatic cirrhosis but has been rarely reported in cardiogenic ascites as well. After an extensive literature review, we were able to identify only seven other cases of this rare entity. Here we present a case of spontaneous bacterial peritonitis developing postoperatively in a 62-year-old male who presented with constrictive pericarditis and cardiogenic ascites initially.

## Case presentation

The patient was a 62-year-old male with a past medical history of coronary artery disease, chronic heart failure, paroxysmal atrial fibrillation on warfarin, recurrent pericarditis, idiopathic constrictive pericarditis, and chronic obstructive pulmonary disease (COPD) that presented to us for evaluation for pericardiectomy. He was asymptomatic on arrival, though he previously had shortness of breath and orthopnea that resolved with outpatient diuretic treatment just before admission. He denied fever, chest pain, further shortness of breathing, or cough. Physical exam was significant for an irregular heart rate, significant lower extremity pitting edema, and abdominal distension with fluid wave consistent with ascites. Consequently, he was admitted to internal medicine service, and cardiothoracic surgery was consulted. Laboratory studies revealed a hemoglobin/hematocrit of 10.8/32 and total iron binding capacity (TIBC) suggestive of anemia of chronic inflammation, and mild elevation of aspartate aminotransferase (AST) at 53 (Ref <41 U/L) with all other liver enzymes within the normal range.

Preoperative testing focused on assessing the possibility of hepatic cirrhosis as an etiology of his ascites, and confirmation of his constrictive pericarditis. Preoperative paracentesis fluid analysis revealed fluid albumin of 2.0 g/dL, a fluid protein of 4.3 g/dL, absolute neutrophil count (ANC) of 13 /cmm, and the serum ascites albumin gradient (SAAG) of 1.4 g/dL (serum albumin 3.4 g/dL). These results revealed a transudative etiology consistent with cardiogenic ascites from heart failure described by SAAG >1.1 and ascitic fluid protein >2.5 g/dL (ascitic fluid protein in cirrhosis is <2.5 g/dL). Subsequent ultrasound of the liver revealed a homogenous liver without nodularity or evidence of cirrhosis. Preoperative right heart catheterization demonstrated features consistent with constrictive pericarditis, including Kussmaul’s sign (a paradoxical rise in jugular venous pressure on inspiration), a “square root sign” on the left ventricular (LV) diastolic tracing, and equalization of LV and right ventricular (RV) diastolic pressures. He then underwent a successful pericardiectomy.

Perioperatively patient developed intermittent fevers ranging from 100 to 101.2 that resolved without any intervention. These fevers were initially attributed to pericarditis. The patient had no leukocytosis and cultures were negative. Postoperatively, the patient developed recurrent ascites requiring a repeat diagnostic/therapeutic paracentesis. Albumin, fluid was 1.3 g/dL, protein, fluid was 3.0 g/dL, serum albumin was 2.1 and ANC was 499 /cmm. Doppler ultrasound did not show any evidence of portal or hepatic vein thrombosis. These findings were consistent with spontaneous bacterial peritonitis and the patient was treated empirically with a five-day course of IV ceftriaxone. The patient remained afebrile and asymptomatic throughout three days of antibiotic therapy, so another paracentesis was performed on day three. This revealed a decrease in ANC to 132 /cmm, demonstrating a therapeutic response. Ascitic fluid cultures were negative for aerobic or anaerobic bacteria, likely due to sterilization. The patient completed his five-day course of antibiotics and did not reaccumulate further ascitic fluid while on furosemide and spironolactone. He was subsequently discharged to a rehabilitation facility.

## Discussion

SBP is a common and life-threatening infection of ascitic fluid first described by Krencker in 1907 [3] and further characterized by Conn in 1964 [4]. SBP is most often caused by Gram-negative bacilli but occasionally, Gram-positive cocci like viridans group streptococci have been implicated. Early diagnosis by paracentesis and treatment with third-generation cephalosporins is essential to reduce mortality [1,2,5]. Different subtypes of SBP exist based on the results of ascitic fluid analysis, with classic culture-positive SBP exhibiting ≥250 polymorphonuclear neutrophils (PMN) and positive culture results. However, if culture results are negative (as in our case), culture-negative SBP can be diagnosed based on a PMN count ≥250. This phenotype can arise secondary to preceding antibiotic therapy, low opsonic characteristics of the ascites, or poor sampling technique [6]. Since our patient received antibiotics intra-and-post-operatively, it is highly likely that our results were affected by sterilization of the causative organism.

SBP is most often seen in patients with liver cirrhosis, though rare reports have described SBP occurring secondary to cardiogenic ascites [7-11], renal ascites [12], and portal vein thrombosis [13], among other rare causes. Bacterial infection of cardiogenic ascites is especially rare, with only seven cases reported to date [7-11]. This rarity is likely secondary to the high protein and opsonic character of cardiogenic ascites, which develops due to the elevated intrahepatic vascular pressures seen in congestive hepatopathy [14]. Simultaneously, however, chronic congestive hepatopathy is thought to cause chronic intestinal damage which predisposes patients to translocation of intestinal bacteria during acute exacerbations. This may be particularly relevant for patients with exacerbations of severe cardiac disease, who may develop hemodynamic instability resulting in intestinal hypoperfusion which dramatically alters gut morphology and the microbiome in ways that may precipitate SBP [11,14]. In our patient, the post-pericardiectomy emergence of SBP suggests that the hemodynamic stress of surgery likely precipitated an acute translocation event possibly through this mechanism.

## Conclusions

Cardiogenic ascites is an uncommon cause of ascites, arising secondary to congestive hepatopathy. Bacterial infection of this fluid is exceedingly uncommon but may occur due to changes arising from intestinal hypoperfusion exacerbated by hemodynamic instability. To our knowledge, this case of SBP of cardiogenic ascites following pericardiectomy in a patient with a history of chronic constrictive pericarditis is the eighth such reported case of SBP in literature. Careful diagnosis with a high clinical suspicion is essential for the timely management of this potentially fatal entity.
